# The Efficacy of Tele-Monitoring in Maintaining Glycated Haemoglobin Levels in Patients with Type 2 Diabetes Mellitus: A Systematic Review

**DOI:** 10.3390/ijerph192416722

**Published:** 2022-12-13

**Authors:** Hope Emonena, Omorogieva Ojo

**Affiliations:** 1 Woodlands Health Centre, 4, Edwin Hall Place, Hither Green Lane, London SE13 6RN, UK; 2 School of Health Sciences, Avery Hill Campus, University of Greenwich, Avery Hill Road, London SE9 2UG, UK

**Keywords:** type 2 diabetes, glycaemic control, tele-medicine, diabetes education, primary care, HbA1c, cost effectiveness

## Abstract

Background: It is well documented that telemedicine offers effective accessibility and consistency which are useful in overcoming the barriers associated with the traditional delivery of chronic disease management. Furthermore, home-based telemonitoring approach for managing chronic disease conditions has been shown to break geographical barriers and facilitate provider-to-patient communication. However, the efficacy of telemedicine in reducing HbA1c is debatable. Aim: This systematic review aims to evaluate the effect of telemedicine on glycaemic control in patients with type 2 diabetes. Method: This systematic review has been conducted in line with Preferred Reporting Items for Systematic Reviews and Meta-Analyses (PRISMA) framework. Searches were primarily conducted using the EBSCOhost database. Other search engines such as Cochrane Library and Google scholar were also used and search of grey literature was performed using google, NHS.uk website, WHO websites, and gov.uk website. Nine articles were included in this review. Results: Three themes were identified in this review including diabetes education/telemonitoring technology and glycaemic control, the attitude of participants, and cost effectiveness of tele-medicine. While three studies reported improved glycaemic control with statistically significant improvement in HbA1c compared to the control group, three other studies did not find significant improvement in glycaemic control. In addition, the findings suggest that participants’ positive attitude to self-care can lead to an improved HbA1c, and finally, several of the selected studies found that telemonitoring is not cost-effective. Conclusion: The findings of this review show that telemedicine may be effective in managing blood glucose in patients with type 2 diabetes. However, factors such as educational level of patients, attitude and costs may limit its application in primary care. More studies are required to fully establish the effectiveness of Telemonitoring in managing patients with type 2 diabetes.

## 1. Introduction

It is well established that people of South Asian and Afro-Caribbean descent are at higher risk of developing type 2 diabetes compared to Caucasians [1,2] and that these patients are predominantly managed in primary care by General practitioners (GPs) and the nursing team. The World Health Organisation [3] describes Diabetes Mellitus as a chronic, metabolic disease that is characterized by chronic hyperglycaemia which leads over time to the risk of micro- and macro-vascular damage. Type 2 diabetes mellitus (T2DM) is regarded as the most common type of diabetes [3] as about 90–95% of people diagnosed with diabetes usually have type 2 diabetes [4]. T2DM is a multifactorial disease that occurs due to the body’s inability to produce enough insulin and/or a resistance to the action of insulin. This tends to develop over time and it is largely lifestyle related [5]. However, there are other risk factors that have been implicated in the development of type 2 diabetes including ethnicity, family history, and specific gene mutations [6].

### 1.1. Diabetes and Its Impact

According to the WHO [3], approximately 422 million people have diabetes worldwide with many of these people living in low- and middle-income countries. The number of people diagnosed with diabetes mellitus has increased significantly in the past years. Davies et al. [7], reported that the rise in Diabetes ranged from 1.4 m in 1996 to 3.5 m in 2014 and most of these people are diagnosed with type 2 diabetes. Whicher et al. [8], also found that seven percent of the United Kingdom (UK) population are living with this condition. Diabetes UK [9] reports that diabetes has resulted in five hundred and thirty myocardial infarctions and a hundred and seventy-five amputations every week and estimates that twenty-four thousand people with diabetes die prematurely due to suboptimal management of the disease with one in six hospitalized. With all these findings and statistics, it is fair to say that the significance of metabolic control cannot be overemphasized as Cavero-Redondo et al. [10] in their study found that patients with elevated levels of glycated haemoglobin (HbA1c) are at a higher risk of cardiovascular disease which results in complications and high costs of prescriptions.

To manage this condition, the UK government sets programmes in place, aimed at preventing the disease as well as improving the outcome of those affected by the condition. Such programmes include the National Health Service Diabetes Prevention Programme (NHS DPP), Diabetes Education and Self-Management for Ongoing and Newly Diagnosed (DESMOND), and Dose Adjustment For Normal Eating (DAFNE) as well as the Quality Outcome Framework (QOF) [11,12,13]. The NHS DPP, DESMOND, and DAFNE are NHS organization programmes that help to deliver high-quality patient education to people with type 1 diabetes mellitus (T1DM) or T2DM or those at risk of the disease. 

QOF on the other hand is a programme specific to primary care providers. It is a pay for performance scheme in the UK and adequate control of glycated haemoglobin is a requirement for primary care [14].

### 1.2. Why It Is Important to Do This Review

The approaches to service provisions have changed drastically since the COVID-19 pandemic in the UK general practice for nurses and other healthcare professionals. With respect to nurses in general practice, the mode of working, pre COVID-19 was predominantly face-to-face with patients being monitored quarterly, six-monthly, or annually based on their level of metabolic control. However, during this pandemic and in planning for post- COVID-19, to address increased treatment needs and to prevent unnecessary in-person contact, patients are now being reviewed remotely. This is also highlighted in the changes made to the Quality Outcome Framework [15].

Homeniuk and Collins [16], conducted a cross-sectional population survey and found that face-to-face consultations in general practices have significantly decreased and concluded in their report that the decrease in face-to-face consultations is likely to continue. It was further reported that the way general practice is delivered will not return to what it was before the COVID-19 pandemic with the public advised to expect increased telemedicine being the norm for the future [16]. 

Since the pandemic, there has been a digital revolution [17]. According to Rodriguez [18], telemedicine is perhaps one of the most significant changes to occur in health care as a result of the COVID-19 pandemic. However, it would appear that there are divergent views on the effectiveness of Telemedicine on health outcomes. For example, Landes et al. [19], identified multiple benefits when dialectical behaviour therapy was provided via telehealth in a stroke unit. Adaji et al. [20], and Zhai et al. [21], also found in their literature review that telemedicine is promising in facilitating self-management of T2DM. However, on the contrary, Wootton [22], is of the view that the value of telemedicine in managing long-term conditions is weak and contradictory. This corroborates the findings of Jaana and Pare [23], who concluded that the magnitude of the effects of telemonitoring on reducing HbA1c is debatable.

One can argue that there is limited evidence regarding the efficacy and practicality of telemedicine in the management of T2DM. Although Kaur et al. [24], found in their study that this intervention can be useful to improve follow-ups and the management of patients with diabetes, it is worthy to note that T1DM were included in this study and there was no mention of the number of T1DM as compared to the T2DM participants. In addition, patients with T1DM are conversant with the technologies utilized in telemedicine because these patients acquire self-management skills at an early age while T2DM is frequently associated with the older population [3]. According to Zhai et al. [21], patients with type 2 diabetes are often unfamiliar with and averse to the technologies used in telemedicine. On the other hand, Hailey et al. [25] indicated in their systematic review that good-quality information on the efficacy, effectiveness, and cost-effectiveness of telemedicine is limited. It is evident that there are issues here that need to be addressed. It is imperative that we understand the impact that this rapid shift towards digital technology has been on clinical practice [26], and assess healthcare providers and patient experiences to ensure continued high-quality care and patient safety.

Aim: To examine the efficacy of tele-monitoring in maintaining glycated haemoglobin levels in patients with type 2 diabetes. 

## 2. Methods

This systematic review has been conducted in line with Preferred Reporting Items for Systematic Reviews and Meta-Analyses (PRISMA) framework [27]. The research question was developed using the Setting, Perspective, Intervention, Comparison, Evaluation (SPICE) framework as a guide. It provides a means of formulating questions to find available evidence in existing research [28].

### 2.1. Types of Studies

Due to the focus of this review, a range of studies including pilot studies and randomised controlled trails (RCT) were included. 

### 2.2. Participants and Interventions

Participants were patients with T2DM on diet control, oral therapy and treated with insulin. Some of the studies included evaluated the impact of their intervention on blood pressure, quality of life, and Lipids profile.

### 2.3. Outcomes of Interest

Primary Outcome Measures: Glycaemic control including glycated haemoglobin levels.

Secondary Outcome Measure: Cost of Telemedicine.

### 2.4. Search Terms and Search Strategy

The main Search engine used was EBSCOhost. EBSCOhost is a valuable resource for health care professionals, and hosts multiple search engines/databases on the web. These include CINAHL plus, Medline, APA PsycINFO, etc. Other search engines used for searches in this review included Cochrane Library as well as Google scholar. Furthermore, a brief search of grey literature was performed via google NHS.uk website, WHO websites, and gov.uk website. 

Additionally, the reference list of published articles was explored. Several keywords, phrases, and Boolean operators were used in order to reduce the number of non-related data/research. These search terms included: T2DM, efficacy, Tele-monitoring, telehealth or telemedicine, type 2 diabetes mellitus, diabetes mellitus, randomized control trials, RCT, glycaemic control, HbA1c. The Boolean operators (and, or, not, and/or) were used to either combine or exclude keywords in the search. Searches were conducted by H.E. and reviewed by O.O.

### 2.5. Inclusion and Exclusion Criteria

The searches were conducted between December 2020 to August 2021. In order to ensure that the most recent articles on this topic were included, only articles published in the last 15 years were selected. Furthermore, as the central focus of the review was based on evaluating the efficacy of tele-monitoring in patients with type 2 diabetes in Europe, North America, Australia, and Canada, only studies published in these areas were included. The inclusion and exclusion criteria are outlined in Table 1. 

**Figure 1:**
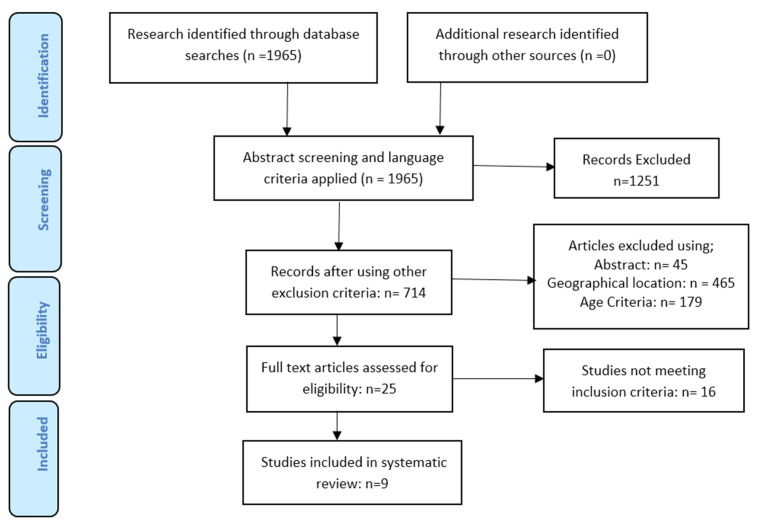
PRISMA Flow Diagram on selection and inclusion of studies.

Based on the first search attempt, 1965 hits were obtained which was then reduced considerably when the fifteen years selection window, English language, and geography were applied. It was further reduced to 25 after reviewing the abstracts to ensure adherence to the inclusion and exclusion criteria and a final total number of nine papers was arrived at after reading through the selected papers. The PRISMA flow chart (Figure 1) outlines the process of selecting the 9 articles included.

### 2.6. Data Extraction

Data were extracted from the studies included by H.E. and crosschecked by O.O. (Table 2).

## 3. Results

Of the nine studies selected for this review, six studies were conducted in the United States, whilst one study each was conducted in France, United Kingdom, and Canada (Table 2). The studies were a mixture of pilot study, observational study, and Randomized controlled trials.

Assessment of Risk of Included Studies.

**Figure 2:**
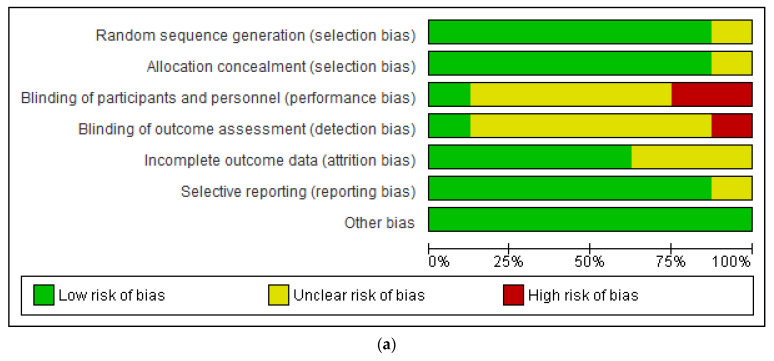

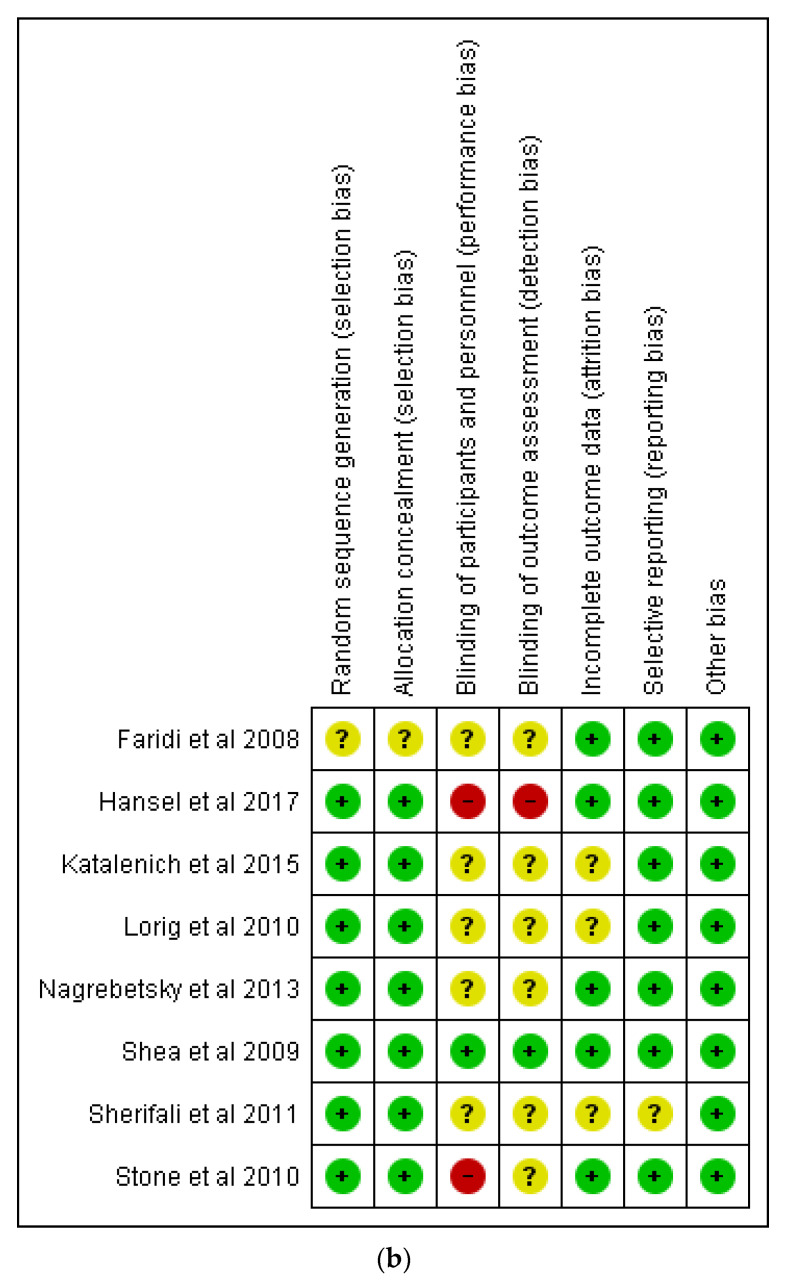
(**a**,**b**): showing risk of bias graph (**a**) and risk of bias summary (**b**) of included studies [29,30,31,33,34,35,36,37].

The eight randomised controlled studies were evaluated using the domain based evaluation tool [38] (Figure 2a,b). The domains assessed were selection bias, performance bias, detection bias, attrition bias, selective reporting and other bias. One study each showed unclear risk of bias for random sequence generation, allocation concealment and selective reporting. While three studies were found to have unclear risk of bias in relation to incomplete outcome data, there were five studies with unclear risk of bias in blinding of participants and personnel, and six studies in blinding of outcome assessment. There was high risk of bias in one study regarding blinding of outcome assessment and 2 studies with high risk of bias in respect of blinding of participants and personnel. The only observational study was assessed using the risk of bias in non-randomized studies of interventions (ROBINS-I) risk assessment tool [39]. There was low risk of bias in all the domains assessed.

Based on the findings of the studies included, three themes emerged in relation to telemedicine that had an impact on glycaemic control.

The identified themes were;

- Diabetes education/Telemedicine and Glycaemic control
- The attitude of participants and glycaemic control
- Cost effectiveness of Telemonitoring

### 3.1. Diabetes Education/Telemedicine and Glycaemic Control

Six studies evaluated the impact of diabetes education and telehealth technology on glycaemic control. Of the six studies, Faridi et al. [29], Nagrebetsky et al. [34] and Sherifali et al. [36] found no statistically significant improvement in HbA1c between the treatment group and the usual care group. In the Faridi et al. [29] study, they reported that the usual means of diabetes care was preferred over the Novel Interactive Cell-phone technology for Health Enhancement (NICHE) system. In contrast, three studies [32,35,37] reported improved glycaemic control with statistically significant improvement in HbA1c compared to the control group.

### 3.2. The Attitude of Participants

Four studies [29,30,33,36] evaluated the impact of the attitude of participants on glycaemic control. All the studies reported an improvement in HbA1c but of minimal effect with no statistical significance. 

The effectiveness of treatment of a disease depends on the level of compliance of the patient with the treatment [40]. Additionally, inadequate compliance with prescribed treatment plans is deemed the most serious obstacle to achieving successful therapeutic outcomes [41,42]. Thus, non-compliance by patients with diabetes is no exception [43]. 

In the NICHE pilot study and the randomized study by Lorig et al. [33], the attitude of the participants could be deduced from the report as there was poor uptake with five participants not transmitting information and poor adherence to the treatment.

In the study by Lorig et al. [33], a good turnout rate was recorded with eighty-one percent of participants completing the trial. However, a high proportion of the participants that joined the programme was actively seeking information about their disease. This is also similar to the RCT by Sherifali et al. [36] where a low dropout rate was recorded. 

### 3.3. Cost Effectiveness of Telemonitoring

Seven studies evaluated the cost effectiveness of telemonitoring on glycaemic control and several of the studies reported that telemedicine is expensive to initiate/install.

In the study by Klug et al. [32], Nagrebetsky et al. [34] and Stone et al. [37], HbA1c improved with statistical significance reported in the Klug et al. [32] study. However, the cost-effectiveness was highlighted as an implication for practice. They reported the technology was expensive to install. This can be seen as a barrier to implementing telemedicine, especially in this era of cost-effective healthcare.

However, the RCT by Hansel et al. [30], revealed that telemedicine has potential to be cost-effective. However, this should be interpreted with caution as no training was required by the participants. In the RCT by Katalenich et al. [31], there was also the potential for the cost-effectiveness of the technology. It is worthy to note that participants’ cell phones and landlines were used to deliver messages. In the RCT by stone et al. [37], participants were required to have a land-based analog home telephone line to be able to join the study. The cost implication of these were not included in the study. Therefore, one can argue that the actual cost of implementing telemedicine was not fully evaluated. 

## 4. Discussion

The findings of this systematic review showed that telemedicine is effective in managing glycaemic control in patients with type 2 diabetes although some of the studies revealed that the improvement in glycaemic control was not significant. Furthermore, educational intervention or a higher educated population is paramount to the successful implementation of telemonitoring for improvement in HbA1c. The findings also suggest that participants’ positive attitude to self-care can lead to an improvement in HbA1c, and finally, several of the selected studies for this review found that telemonitoring is not cost-effective.

### 4.1. Education, Telehealth Technology and Glycaemic Control

Education for both participants and providers is imperative in any intervention. This was made clear in the United Kingdom (UK) National Health Service (NHS) report by the Commission on Education and Training for Patient Safety [44]. Self-management is an important part of effectively managing diabetes patients and to equip patients to be independent in this role, they need to be educated. Therefore, telemonitoring should not be an exception. This was evident in all the studies included in this review as they reported educational intervention or participants’ level of education in the studies. Diabetes education is an ongoing process of facilitating the knowledge, skill, and ability necessary for diabetes self-care and it is necessary to improve patient outcomes [45]. The American Diabetes Association [46] recommends continuing diabetes education. Additionally, the Healthy People Programme [47] was aimed at increasing the proportion of individuals with diabetes who receive formal diabetes education. Hence, the impact of diabetes education cannot be underestimated. 

Although, the selected research emphasizes that education aids telemonitoring in improving HbA1c, in some of the research, the improvement was not statistically significant. However, it is worthy to note that there was no description of the type of educational intervention employed in those studies that reported no statistically significant improvement in HbA1c. Therefore, this makes it challenging to evaluate the findings of those papers. 

In a systematic review by Van-Dam et al. [48], it was shown that recent studies indicate that an important aspect of diabetes care is the active involvement of patients in their care and an avenue to achieve effective outcomes is to use patient education to facilitate the learning process of self-management. The relationship between understanding HbA1c and diabetes self-care behaviors was shown by Beard et al. [49]. In their study, they found out that seventy-eight percent of the participants had poor knowledge and understanding of HbA1c. They then went further to recommend the introduction of programs and initiatives such as (DESMOND), as improving self-efficacy may in turn improve clinical outcomes. Similarly, Siminerio et al. [50] also recommended diabetes education after they found out in their observational study that a significant barrier to achieving optimal glycaemic control by participants was removed with access to a trained nurse to provide education and support to the participants. 

Interestingly, the systematic review by Gerrald et al. [51], found no benefit of telemonitoring in improving HbA1c and reported that HbA1c was unaffected by education aimed at self-management and study follow-up. This was also the case in some of the selected papers for this systematic review that reported no statistically significant benefit of education in improving HbA1c. However, the contents of the educational intervention, phone calls as well as details of prior knowledge of the participants were not made available for analysis. 

### 4.2. Attitude of Participants

The impact of patients’ attitudes on any outcome can be very significant. This is because they are the ones that will effectively implement the agreed care plan. Therefore, a person’s attitude towards their self-care greatly impacts their commitment to the agreed care plan and the overall outcome of the intervention. The power of commitment in influencing change cannot be underestimated. Argyris [52] advises that only internal commitment reinforces empowerment and to understand why there is no transformation, we need to start with commitment. 

One major factor that impacted participants’ attitudes is their educational background/level of education. As mentioned previously, Millar et al. [53], in their cross-sectional study reported that education was found to motivate the patients to change their dietary and physical activity behavioural patterns which will invariably reduce HbA1c in patients with diabetes. The issue of commitment and/or positive attitude was evident in the selected research for this review as it impacted the attrition rate as well as the HbA1c. 

From these studies, it is clear that the participants were relatively well-educated, and the sample may have over-represented participants who were motivated and actively engaged and/or seeking information about their diabetes care. We believe that the findings from these studies may be difficult to implement in communities where people have a low level of education and/or from other cultures that may have different needs that were not explored in this study.

Research conducted on telemonitoring on quality of life found that it results in depressive symptoms, greater levels of distress, and anxiety [54,55] which may impact on compliance rate. This adds to the controversy because the frequency of follow-up was found to improve outcomes in the systematic review by Woods et al. [56]. Additionally, Heath [57] argues that since patients spend more time outside the clinic than in it, providers should keep communication routes open and make sure patients remain activated in their care. Heath [57] went further to highlight that one way of achieving this is via telemonitoring. It would not be unreasonable to expect that any research aimed at patient adherence to treatment plan would consider the impact of the follow-up frequency and therefore, ensures measures are in place to determine patients’ adherence to the agreed care plan.

It is well documented that only seven percent of patients with diabetes are adherent to all aspects of their treatment plan [58]. Becker and Janz [43] argue that this may be somewhat based on the realization by patients that adherence does not necessarily lead to a lack of illness and Beard et al. [49] argue it may be that patients have a contrasting interpretation of their diabetes management. Hence, there is a need to better understand how psychosocial factors impact adherence behaviours [59]. 

### 4.3. Cost Effectiveness of Telemonitoring

It is not possible to completely isolate the issue of cost when dealing with telemonitoring. Several of the selected studies for this project found that telemonitoring may not be cost-effective. Therefore, cost is a major factor to consider when drawing up protocols for any chronic disease management. The findings of this review agree with Henderson et al. [60], in their cluster randomized controlled trial where they noted that telehealth was found not to be a cost-effective means of managing patients with long-term conditions. 

Time and personnel are needed to follow up with patients either virtually or face-to-face. Therefore, adopting telemedicine linked with face-to-face appears to be the way forward in diabetes management. This is because, in practice, patients only need to be seen face-to-face for clinical procedures such as diabetes foot checks, blood pressure, measurement of body mass index, and blood test. 

In most of the selected papers for this review, the incidence of clinical outcomes was similar in both the usual care and intervention groups. Therefore, the cost may be the deciding factor when having to choose which method of monitoring to adopt because according to Jones-Devitte and Smith [61], practices must not only be effective, but they also need to be viable economically. In this era of cost-effective healthcare, every effort should be made to provide clear guidelines regarding the frequency of follow-up in this client group. 

The guideline for monitoring patients with diabetes is not set in stone. According to the National Institute for Health and Care Excellence [62], a minimum of one follow-up annually is recommended with quarterly follow-up for poorly controlled diabetes. However, this is only a recommendation and appears to contrast with what happens in practice, where patients with poorly controlled diabetes are often seen face-to-face every four weeks to six weeks. In the study by Katalenich et al. [31], the frequency of calls and face-to-face visits positively impacted HbA1c. This indicates that telemonitoring should complement face-to-face visits. It should be noted that subject heterogeneity makes follow-up regimen variable because every patient’s clinical circumstances are different. 

NICE [63] recommends that decisions on care provision should be based on the best available evidence of both the cost and clinical effectiveness. This is also one of the principles of prescribing whereby prescribers are required to select safe and cost-effective medicines [64]. While providers of care may evaluate changes with the focus not only on safety but on reducing cost, the patients’ focus is mainly geared towards effective outcomes notwithstanding the cost to providers, especially in a system like the NHS where care is free for permanent residents [65] and most patients are not faced with private health insurance bills to consider. In one out of the seven studies reviewed under this theme, telemonitoring was reported to be cost-effective. This may not be a true reflection of the cost implication as a rigorous cost analysis was not performed because, it is evident that with no training requirements by the participants and participants were required to have their telemonitoring equipment, this cuts out the huge cost of training and equipment as well as installation and subscription charges for maintaining the equipment. Therefore, the consensus is that telemonitoring is expensive to initiate and maintain [66].

### 4.4. Implication for Future Practice

Patients with type 2 diabetes need to be supported to self-manage their condition using an evidence-based approach. This empowers patients to make informed decisions about their health care and gain control of their diabetes and Telemedicine has been identified as a feasible/possible means of providing this support to patients with type 2 diabetes. Telemedicine can be achieved through multiple approaches such as telephone consultations, text messaging, online programmes. The use of telephone as a strategy for telemedicine may make this intervention cost-effective for adoption in healthcare practice as most patients can be contacted via telephone be it mobile or landline. This excludes the barrier that may be caused by a lack of experience with computers and/or internet use by patients.

Based on the findings of this review, it has been shown that education is useful in promoting uptake and impacts positively on the attitude of patients with T2DM thereby improving clinical outcomes. However, the evidence from the literature reveals that there is no unanimity on the cost-effectiveness of Telemonitoring. More research is needed to explore and evaluate the cost-effectiveness of telemedicine in supporting patients with T2DM to manage their HbA1c. 

### 4.5. Limitations of This Review

One key strength identified in this systematic review is that it is in line with the current climate of change in health care provision as it explored the impact of telemedicine on HbA1c control considering there is a push for telemedicine in chronic disease management with the emergence of COVID-19.

However, the limitation of the review is that it is aimed at telemedicine in general rather than focusing on an aspect of telemedicine such as telephone calls, text messages, web interactions, etc., therefore limiting the generalization of the findings. This is because the cost involved in using mainly telephone calls may differ from other approaches that require landlines or analogue installations. This also has an impact on the level of education and/or training as well as equipment cost that may be required with an online-based approach.

Furthermore, the potential bias with respect to the blinding of participants and personnel, and outcome assessment in some of the studies included may affect the quality of the systematic review.

## 5. Conclusions

Based on the findings of this review, it has been shown that although telemedicine may be effective in managing blood glucose in patients with type 2 diabetes, factors such as educational level of patients, attitude and costs may limit its application in primary care. Furthermore, the efficacy of Tele-monitoring in maintaining HbA1c in patients with T2DM, may be enhanced when combined with face-to-face contacts and appropriate interventions such as medication as well as dietary reviews. More studies are required to fully establish the effectiveness of Telemonitoring in managing patients with type 2 diabetes.

## Figures and Tables

**Table 1:** Inclusion and Exclusion Criteria.

Inclusion Criteria	Exclusion Criteria
Adults: Age 18 years to 75	Age under 18 s and over 75 s
Research that is written in English and not older than fifteen years	Research that is written in a foreign language with the age of research older than fifteen years old.
T2DM patients on diet control or treatment (oral therapy or insulin)	T1DM, Gestational diabetes, Maturity Onset Diabetes of the Young (MODY).
Primary/main research, focus groups and pilot studies.	Systematic reviews, meta-analyses, letters, case reports, guidelines, editorials, and technical reports.
Countries in North America, Europe, Canada and Australia.	Countries in Asia, Africa, and South America.

**Table 2:** Characteristics of studies included in the review.

Authors and Country of Study	Aims and Objectives	Research Methods	Sample Size, Demography and Duration of Intervention.	HbA1c (Baseline, Final Value and Change)	Main Findings	Implications for Practice
Faridi et al. [29] USA	To examine the feasibility and utility of the NICHE technology to assist with diabetes self-care in a clinic population as well as its impact on clinical outcomes.	Phase 1 pilot- Study. Randomised Controlled Trial	*n* = 30 T2DM diagnosed at least one year T2DM on a diet or oral therapy. Age, greater or equal to 18 years with a Mean age of 55.3 years BMI > 25 No insulin usage. Duration of Intervention: 3 months Female (Intervention): 60% Male (Intervention): 40% Female (Control): 66.7% Male (Control): 33.3%	HbA1c: Mean ± SD Baseline Intervention: 6.4 ± 0.6% Baseline Control: 6.5 ± 0.7% Change Intervention: −0.1 ± 0.3% Change Control: 0.3 ± 1.0%	HbA1c improved. However, not statistically significant. Positive trends in the utility of the technology suggest it has the potential to improve glycemic control. Usual means of diabetes care are preferred over the NICHE system.	A larger and longer intervention and evaluation are warranted. However, only after the technology is upgraded and improved.
Hansel et al. [30] France	Evaluate fully automated web-based intervention designed to help users improve their dietary habits and increase their physical activity.	Randomised Clinical Trial	*n* = 120 Age: 18–75 years with mean age of 57 ± 9 years Women= 80/120 (66.7%) Men: 33.3% Mean BMI 33 kg/m^2^ Duration of Intervention: 4 months	Mean Difference [95% CI] Intervention: −0.37 (1.04) Mean Difference [95% CI] Control: 0.23 (0.73)	Trial improved dietary habits. Glycaemic control also improved. Can be delivered remotely with limited human resources and therefore has potential for cost-effectiveness.	Difficult to generalize due to the relatively small sample size. Feasibility of implementation would be easy in a population that is accustomed to using the internet.
Katalenich et al. [31] USA	Evaluate the hypothesis that an automated system such as the DRMS is as effective in achieving improvement in glycemic control and management as in usual clinical care.	Randomised control study. Two = arms randomization intervention and control groups.	*n* = 98 T2DM on insulin and oral therapy. Highly educated with a high school diploma or higher. Greater or equal to 18 years. Female (Intervention): 34 (68%) Male (Intervention): 16 (32%) Female (Control): 25 (52%) Male (Control): 23 (48%) Duration of Intervention: 6 months	HbA1c: Median Baseline Intervention: 8.35% Baseline Control: 8.30% 6 months Intervention: 8.1% 6 months Control: 7.9%	HbA1c was similar between the DRMS and control group at 3&6 months.	Not suited to more complex insulin adjustment algorithms.
Klug et al. [32] USA	To explore the feasibility of incorporating a telehealth system into an existing telephone diabetes management program utilizing a clinical pharmacist.	Observational study.	*n* = 28 Predominantly T2DM No nursing home or hospice patients. Age: >18 years, mean age of 50 ± 13.4 years Female: 78.6% Male: 21.4% Duration of Intervention: 4 months	HbA1c: Mean ± SD Baseline Intervention: 9.8 ± 2.08% Post Intervention: 8.5 ± 2.20%	Mean blood glucose decreased significantly *p* = 0.0002 Telehealth technology can be a positive adjunct to the primary care team in managing diabetes or other chronic conditions to improve clinical outcomes.	Expensive to install (funding).
Lorig et al. [33] USA	The hypothesis that people with T2DM in an online self-management program compared to usual care would demonstrate reduced HbA1c at six and eighteen months…	Three-arms randomized study 2/3 treatment group, subdivided into follow-up and no follow-up 1/3 usual care	*n* = 761 Average age 54.3 years Predominantly non-Hispanic whites 76% Age = 18 years and over 110 American Indian/Alaska natives. Married 66% Average age: 54.3 years Female: 73% Male: 27% Duration of Intervention: 18 months	HbA1c: Mean ± SD Baseline Intervention: 7.12 ± 1.59% Baseline Control: 6.71 ± 1.25% Change Intervention: −0.088 ± 1.24 Change Control: 0.206 ± 0.973	It may have beneficial effects in reducing HbA1c. At 6 months improvement in HbA1c was significant in the intervention group compared with usual car (*p* < 0.05). The follow up reinforcement had no value.	The program may be more suited to patients with higher HbA1c. Further study is required. Increased likelihood of HbA1c getting worse due to participants’ low HbA1c at baseline which indicated patients were in control. However, the poorly controlled patients with HbA1c >7% achieved a significant reduction of *p* = 0.01
Nagrebetsky et al. [34] UK	Feasibility of stepwise self-titration of oral glucose-lowering medication guided by a mobile telephone-based telehealth platform for improving glycemic control in type 2 diabetes.	Open parallel group randomized control feasibility study in primary care.	*n* = 14 T2DM on oral treatment 35 years old or greater. Mean age: 58 ± 11 years. Mean BMI 32.9 kg/m^2^ Male: 71% Female: 29% Caucasian participants only. Duration of Intervention: 12 months	HbA1c: Mean ± SD (mmol/mol) Baseline Intervention: 64 ± 11 Baseline Control: 66 ± 13 HbA1c: Median ± (IQR) (mmol/mol) Change Intervention at 6 months: −10 (−21 to 3) Change Control at 6 months: −5 (−13 to 6)	Self-titration of oral glucose-lowering medication in T2DM with self-monitoring and remote monitoring of glycemic control is feasible. Further studies using adapted recruitment strategies are required to evaluate whether it improves clinical outcomes.	Expensive to initiate. The generalization of findings was limited because participants were limited to one ethnic group only.
Shea et al. [35] USA	Examine the effectiveness of telemedicine interventions to achieve clinical management goals in older, ethnically underserved patients with diabetes.	Randomized controlled trials.	*n* = 1665 55 years and older Mean Age (Intervention): 70.8 ± 6.5 years Mean Age (Control): 70.9 ± 6.8 years Diabetic on treatment with diet, oral supplements, or insulin Fluency in English or Spanish. 15% African Americans, 35% Hispanics 63 women Lower socioeconomic and educational levels, and low levels of computer literacy. Female (Intervention): 63.5% Male (Intervention): 36.5% Female (Control): 62.1% Male (Control): 37.9% Duration of Intervention: 5 years	HbA1c: Mean ± SD (%) Baseline Intervention: 7.36 ± 1.48 Baseline Control: 7.40 ± 1.60 Final Intervention: 7.05 ± 1.17 Final Control: 7.34 ± 1.54	Telemedicine case management resulted in a net improvement in HbA1c relative to usual care. *p* = 0.001 Glycemic control in the usual care group improved during the first two years and then reverted to baseline. Differences were present at one-year follow-up and sustained over five years. Multifactorial improvement has greater clinical significance than improvement in single risk factors.	A high likelihood of generalization due to the ethnical diversity and enrollment in primary care practices.
Sherifali et al. [36] Canada	To find out whether computer-generated patients’ feedback leads to improvement in glycemic control in people with T2DM after one year.	Two-arm open randomized control trial	*n*= 465 (1-1 randomization) Mean Age (Intervention): 62 ± 11 years Mean Age (Control): 62 ± 10 years Female (Intervention): 49% Male (Intervention): 51% Female (Control): 53% Male (Control): 47% Duration of Intervention: 12 months	HbA1c: Mean ± SD (%) Baseline Intervention:7.85 ± 0.88 Baseline Control: 7.81 ± 0.83 HbA1c: [95% CI] Change Intervention at 12 months: −0.24 (−0.37 to −0.12) Change Control at 12 months: −0.15 (−0.27 to −0.03)	HbA1c decreased in both arms but the difference in HbA1c in both arms was not significant. The incidence of clinical outcomes such as hospitalisation and treatment of foot ulcers was similar in both groups.	Quarterly community-based intervention providing computer-generated recommendations may be insufficient to significantly change HbA1c levels. The results of the main findings can be generalized as the study is focused on a particular setting (community-based).
Stone et al. [37] USA	To compare the short-term efficacy of home telemonitoring couples with active medication management by a nurse practitioner with a monthly care coordination telephone call on glycemic control in veterans with type 2 diabetes and entry HbA1c of equal or greater than 7.5%.	A randomized controlled trial (RCT).	*n* = 150 Treatment group = 73 and control group = 77 Mainly veterans Participants in both groups were ≥65 years Female (Intervention): 0% Male (Intervention): 100% Female (Control): 2.7% Male (Control): 97.3% Duration of Intervention: 6 months	HbA1c: Mean ± SD (%) Baseline Intervention: 9.6 ± 1.6 Baseline Control: 9.4 ± 1.4 Final Intervention at 6 months: 7.9 ± 1.2 Final Control at 6 months: 8.6 ± 1.3	Treatment demonstrated a significant reduction in HbA1c with most improvement occurring at three months (*p* < 0.001). Both groups improved their HbA1c.	Cost–benefit analysis recommended Long-term sustenance of benefits (reduced HbA1c) beyond 6 months is uncertain.

Abbreviations: HbA1c (Glycated Haemoglobin); IQR (Inter Quartile Range); NICHE (Novel Interactive Cell-phone technology for Health Enhancement); SD (Standard Deviation); T2DM (Type 2 diabetes mellitus).

## Data Availability

We conducted secondary data analysis of publicly available data.
